# Characterization of the Micromorphology and Topochemistry of Poplar Wood during Mild Ionic Liquid Pretreatment for Improving Enzymatic Saccharification

**DOI:** 10.3390/molecules22010115

**Published:** 2017-01-11

**Authors:** Sheng Chen, Xun Zhang, Zhe Ling, Feng Xu

**Affiliations:** Beijing Key Laboratory of Lignocellulosic Chemistry, Beijing Forestry University, Beijing 100083, China; shengchen@bjfu.edu.cn (S.C.); zhangxunyy@bjfu.edu.cn (X.Z.); jjling814@bjfu.edu.cn (Z.L.)

**Keywords:** poplar cell wall, ionic liquid pretreatment, mild conditions, enzymatic hydrolysis, micromorphology, topochemistry

## Abstract

Ionic liquids (ILs) as designer solvents have been applied in biomass pretreatment to increase cellulose accessibility and therefore improve the enzymatic hydrolysis. We investigated the characterization of the micromorphology and the topochemistry of poplar wood during 1-ethyl-3-methylimidazolium acetate pretreatment with mild conditions (90 °C for 20 and 40 min) by multiple microscopic techniques (FE-SEM, CLSM, and CRM). Chemical composition analysis, XRD, cellulase adsorption isotherm, and enzymatic hydrolysis were also performed to monitor the variation of substrate properties. Our results indicated that the biomass conversion was greatly enhanced (from 20.57% to 73.64%) due to the cell wall deconstruction and lignin dissolution (29.83% lignin was removed after incubation for 40 min), rather than the decrystallization or crystallinity transformation of substrates. The mild ILs pretreatment, with less energy input, can not only enhance enzymatic hydrolysis, but also provide a potential approach as the first step in improving the sequential pretreatment effectiveness in integrated methods. This study provides new insights on understanding the ILs pretreatment with low temperature and short duration, which is critical for developing individual and/or combined pretreatment technologies with reduced energy consumption.

## 1. Introduction

Biofuels are considered to be the main potential replacement for fossil fuels in the near future. Liquid biofuels like bioethanol can be produced via the fermentation of sugars produced from the cellulose and hemicelluloses within the lignocellulosic biomass, such as wood and agricultural or forest residues. These abundant, readily available, and low-cost lignocellulosic materials can capture huge amounts of solar energy and therefore play a crucial role in the development of second-generation bioethanol. However, unlike the starch from corn or other grains that is susceptible to hydrolysis, it is hard to hydrolyze the lignocellulosic biomass enzymatically due to ‘biomass recalcitrance’, the natural resistance of plant cell walls to microbial and enzymatic deconstruction [1]. Therefore, an additional deconstruction step called pretreatment is required to bring the biomass feedstock into a form suitable for hydrolysis and subsequent fermentation [2].

A number of pretreatment technologies have been developed in the past years and reviewed by researchers [2,3,4,5,6]. Kumar et al. compared the advantages and limitations of several pretreatment methods [6]. For example, the alkaline pretreatment can increase accessible surface area and then achieve high hydrolysis rates, but this method requires long residence times and high temperatures. Dilute acid pretreatment was limited by the formation of inhibitors such as acetic acid, furfural, and hydroxymethylfurfural (HMF), which impact the subsequent fermentation. Furthermore, these pretreatment technologies cannot be universally applied to a variety of biomass, especially feedstock with relatively higher lignin content [4,7].

Ionic liquids (ILs) are organic salts that usually melt below 100 °C; they consist of a large organic cation and a small organic or inorganic counterion. Due to their high thermal stability, nearly complete non-volatility, wide liquid range, and excellent solvation properties, ILs are becoming attractive alternatives to volatile and unstable organic solvent [8]. Moreover, with almost a limitless combination of anions and cations that can be used to synthesize ILs, they are often referred to as designer solvents [9,10]. ILs have been already applied in many fields such as synthesis, catalysis, batteries, and fuel cells [11]. In addition, ILs have the potential and promising application in biomass pretreatment for the conversion of fermentable sugar. They are widely applicable to different types of lignocellulosic feedstocks due to their unique ability to dissolve the complete lignocellulosic matrix [12]. Dadi et al. applied ILs to pretreat cellulose for the first time; in that report, the microcrystalline cellulose (MCC) was dissolved and regenerated in 1-butyl-3-methylimidazolim chloride ([Bmim]Cl) [13]. Recently, studies have shown that pretreatment using ILs, such as 1-ethyl-3-methylimidazolium acetate, ([Emim]Ac) can significantly reduce biomass recalcitrance and therefore enhance the enzymatic hydrolysis of fermentable sugar [14,15]. This improvement of biomass conversion efficacy was attributed to the impact of ILs pretreatment on lignocellulose substrate properties; decrystallization or crystallinity transformation from cellulose I to cellulose II, extraction of hemicelluloses and lignin, deconstruction of biomass matrix, and partial reduction in degree of polymerization of cellulose. The effect of ILs pretreatment on substrate properties and the pretreatment efficacy depend on the type of ILs and the pretreatment conditions. To date, due to the higher biomass solubility, minimal impact on the environment, and relatively low toxicity to animals and humans, imidazolium-based ILs, especially [Emim]Ac, have received the most attention [16]. Rogers et al. reported that the [Emim]Ac can completely dissolve both softwood (southern yellow pine) and hardwood (red oak) by heating at 110 °C for 16 h. Then, the appropriate reconstitution solvents were able to regenerate carbohydrate-free lignin and cellulose-rich materials. The authors compared dissolutions of the hardwood and softwood in [Emim]Ac and [Bmim]Cl [17]. In 2010, Schall et al. used [Bmim]Ac to pretreat poplar and switchgrass with 5% biomass loading at 120 °C for 30 min. Using a commercial cellulase system, 85% glucose and 76% xylose yield were obtained from the pretreated poplar, which were greatly higher than those from the untreated substrates [18].

In the previous studies, most of the ILs pretreatment of biomass or cellulose prior to enzymatic hydrolysis or second-step pretreatment in combined methods is performed at a temperature higher than 100 °C and with a long duration [17,18,19]. Lower temperature and shorter incubation time can minimize the energy intake of ILs pretreatment. Although this mild pretreatment processing cannot achieve dramatically high efficiency of enzymatic hydrolysis like other severe pretreatments, the sequential process step in integrated pretreatment technologies or the allowed one-batch processing of ILs pretreatment and saccharification can compensate the loss of pretreatment effectiveness in a way. However, little attention has been paid to ILs pretreatment with mild processing conditions for improving enzymatic hydrolysis, especially for the study of micromorphology and topochemistry of pretreated biomass. Auxenfans et al. studied mild ILs pretreatment and enzymatic saccharification of cellulose towards one-batch process; the structural changes of pretreated cotton cellulose were investigated, and the pretreatment condition was optimized [20]. The structural and topochemical changes of plant cell walls after ILs pretreatment with severe conditions (120 °C for 0.5–3 h) were also studied by confocal laser scanning microscopy (CLSM) and confocal Raman microscopy (CRM) [21]. However, no studies have combined the investigation at wood-flour level and subcellular level to understand the ILs pretreatment with mild conditions and then guide the development of individual pretreatment methods and/or integrated pretreatment technologies, with reduced energy input, for improving enzymatic saccharification.

The objective of this study is to reveal the mechanism of mild [Emim]Ac pretreatment for improving the enzymatic hydrolysis of biomass with less energy input by combing the information about the microstructure and the topochemistry of pretreated poplar wood. Multiple microscopic techniques, chemical composition analysis, powder X-ray diffraction (XRD), cellulase adsorption isotherm, and enzymatic hydrolysis were performed to monitor the variation of substrate properties. We present evidence that cell wall deconstruction and lignin dissolution during mild [Emim]Ac pretreatment play an important role in improving enzymatic hydrolysis. In addition, this mild IL pretreatment with reduced energy intake may improve the sequential (second-step) pretreatment efficacy in integrated pretreatment technologies, which will be further investigated in future work.

## 2. Results and Discussion

### 2.1. Chemical Composition and Enzymatic Hydrolysis

The chemical composition content of biomass samples is a significant factor that affects the sequential enzymatic hydrolysis. The chemical compositions of poplar samples untreated and pretreated with IL are illustrated in Table 1. As can be seen, the raw poplar samples used in this study contained 45.08% cellulose, 19.79% hemicelluloses, and 22.53% lignin, which is similar to the samples reported in the literature [22,23]. Dramatic changes in chemical compositions, especially lignin, were observed in the IL pretreated poplar samples.

As mentioned, ILs can dissolve a large number of biomacromolecules such as cellulose, hemicelluloses, and lignin with high efficiency. These chemicals can be regenerated from solutions by adding anti-solvents such as water and ethanol. [Emim]Ac, one efficient solvent in dissolving wood flour, can remove lignin with relatively good selectivity [24]. Table 1 shows the removal of acid-insoluble lignin and acid-soluble lignin from poplar samples after [Emim]Ac pretreatment. Taking the rate of residue recovery into account, 23.48% and 29.83% lignin was removed from the poplar samples after pretreatment with [Emim]Ac at 90 °C for 20 min and 40 min, respectively. This is not the maxima value of lignin that can be removed by [Emim]Ac, due to the mild temperature and especially short duration. Lee et al. reported that 86% of the lignin was extracted from maple wood after [Emim]Ac pretreatment at 90 °C for 70 h [24].

An increase of the relative cellulose content of pretreated poplar (from 45.08% to 50.91%) was benefited by the extensive removal of lignin. In fact, little carbohydrates were extracted from the wood flour by [Emim]Ac; the content of glucan, xylan and other sugars changed slightly during [Emim]Ac pretreatment with mild conditions.

**Figure 1:**
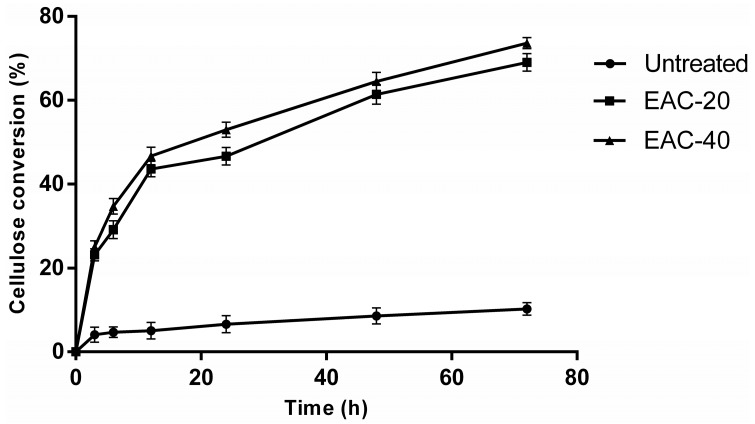
Enzymatic hydrolysis profile of poplar wood untreated and pretreated with [Emim]Ac at 90 °C. EAC-20: [Emim]Ac pretreated for 20 min. EAC-40: [Emim]Ac pretreated for 40 min. Error bars indicate standard deviation.

In order to evaluate the effectiveness of IL pretreatment on cellulose digestibility, the raw poplar samples and the residues after [Emim]Ac pretreatment were subjected to a 72-h enzymatic hydrolysis test. The temporal profiles of cellulose conversion were presented in Figure 1. To avoid the toxic or inhibitory effects of IL on the growth of microorganisms and ensure the validity of comparison, all the samples were washed thoroughly with deionized water and the enzymatic hydrolysis assays were conducted under the same conditions. As can be seen, the [Emim]Ac pretreated poplar samples were readily degraded by cellulases; high cellulose conversions (73.64% and 69.02% for durations of 40 min and 20 min, respectively) were observed after enzymatic hydrolysis for 72 h. This enhancement of enzymatic digestibility for biomass was obvious and significant compared with the cellulose conversion of untreated poplar samples (only 10.26%). Lee et al. [24] reported that the digestibility of maple wood flour after [Emim]Ac pretreatment under similar mild conditions (90 °C, 30 min) was 65%. After increasing the pretreatment severity, a digestibility higher than 95% was achieved. The study performed by Singh et al. [14] showed that 72.5% of switchgrass is converted to sugar for the switchgrass, which indicated that the [Emim]Ac was also effective for improving the digestibility of herbaceous materials.

Lignin reduces the effectiveness of enzymatic hydrolysis by limiting the cellulose accessibility as well as by binding cellulose unproductively. Although the relative contribution of these two roles of lignin is not yet fully understood, the removal of lignin by IL pretreatment in this study has actually improved the enzymatic hydrolysis of poplar samples.

### 2.2. Substrate Accessibility to Cellulase

**Figure 2:**
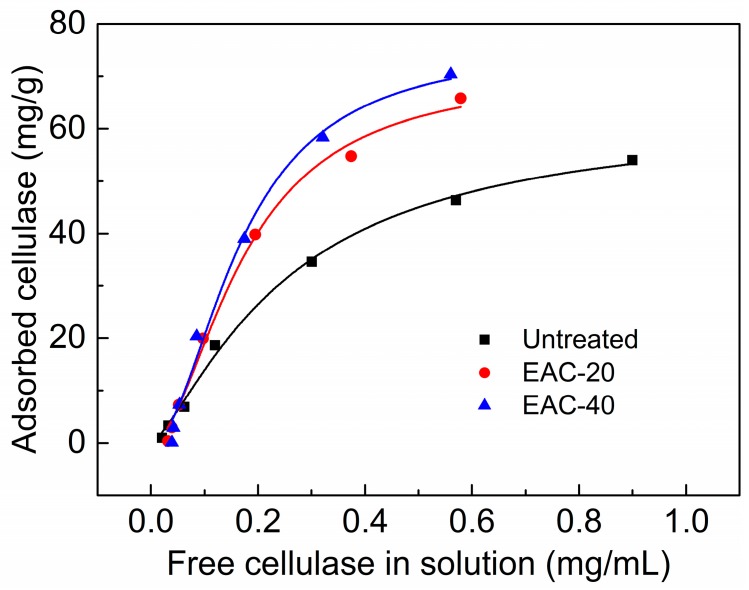
Cellulase adsorption isotherm on poplar wood untreated and pretreated with [Emim]Ac at 90 °C. EAC-20: [Emim]Ac pretreated for 20 min. EAC-40: [Emim]Ac pretreated for 40 min.

The adsorption isotherm of enzyme adsorption on untreated and IL pretreated substrates was determined using cellulase to further address the effect of IL pretreatment on cellulose accessibility. By fitting the adsorption data in Figure 2 to the Langmuir isotherm, the corresponding parameters for the three substrates were obtained and listed in Table 2, along with the determination coefficient (R^2^) in the range of 0.987–0.995. The high R^2^ suggested that the model was adequate for describing the adsorption of cellulase in the present study. As shown in Table 2, the IL pretreated substrate had a higher adsorption capacity (E_max_ = 71.15, 76.15 mg/g) than the untreated substrate (E_max_ = 62.83 mg/g). The Langmuir constant (K_d_) and distribution coefficient (K_r_) can be used to estimate the affinity of enzymes for substrates [26]. The values of these two parameters increased significantly after IL pretreatment, indicating that cellulase had a higher relative affinity for IL pretreated substrates than for untreated substrates. These results further explained the improvement of enzymatic hydrolysis for the IL pretreated samples shown in Figure 1. Goshadrou et al. observed the similar enhancement of binding capacity and affinity for cellulase in IL pretreated wood samples, compared to the untreated materials [27].

### 2.3. Cellulose Crystallinity

**Figure 3:**
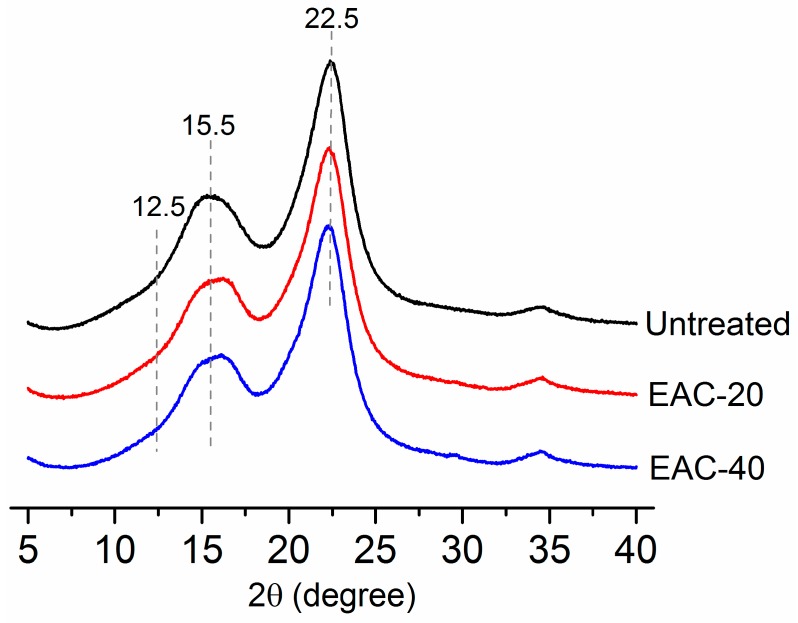
X-ray diffractograms of the poplar wood untreated and pretreated with [Emim]Ac at 90 °C. EAC-20: [Emim]Ac pretreated for 20 min. EAC-40: [Emim]Ac pretreated for 40 min.

Cellulose crystallinity is a key factor that limits cellulose accessibility and then affects the effectiveness of enzymatic hydrolysis. ILs pretreatment with severe processing conditions improves the enzymatic hydrolysis of biomass through decrystallization and/or crystallinity transformation [28,29]. However, in this study, the decline of cellulose crystallinity index was small and the samples kept the crystallinity of cellulose I after [Emim]Ac pretreatment due to the short time and mild temperature. As can be seen from Figure 3, the changes of the cellulose crystalline structure during IL pretreatment were displayed using XRD spectra. The intensity of peaks at 22.5° and 15.5° decreased slightly for [Emim]Ac pretreated poplar samples, compared with that of the untreated samples. The cellulose crystallinity index was calculated by Equation (3) and presented in Table 1; the extent of the crystallinity index (CrI) reduction was negligible (from 39.9% to 38.6%) after [Emim]Ac pretreatment at 90 °C for 40 min. Moreover, a specific peak around 12.5° (reflection 1 1 0, cellulose II) was not observed, indicating the samples still held cellulose I.

Our results indicate cellulose crystallinity is not the only factor that influences the effectiveness of enzymatic hydrolysis of biomass. In addition to the above chemical composition changes, there are other factors affecting the efficient conversion of biomass to fermentable sugars.

### 2.4. Morphological Changes

**Figure 4:**
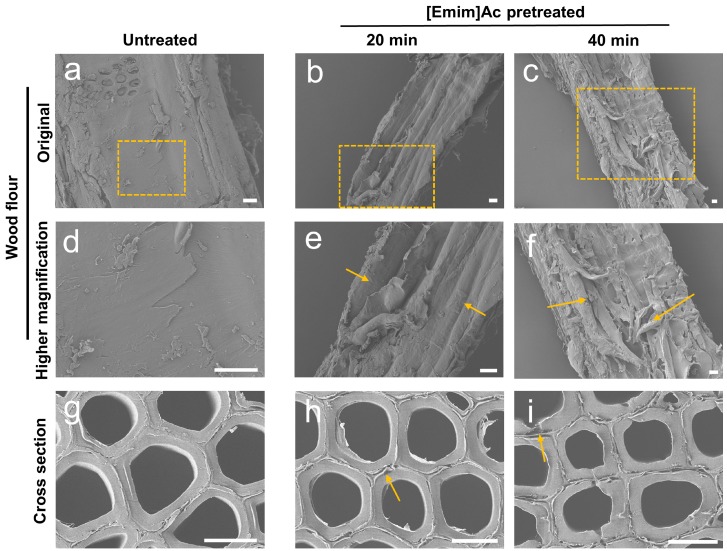
FE-SEM images of raw and [Emim]Ac pretreated poplar wood at 90 °C. (**a**–**f**) wood flour images; Images (**d**–**f**) are the higher magnification of the regions boxed in (**a**–**c**); Arrows in (**e**), (**f**) indicate cell wall deconstruction; (**g**–**i**) cross section images. Arrows in (**h**), (**i**) indicate lamella detachment and gaps. Scalebar = 10 μm.

**Figure 5:**
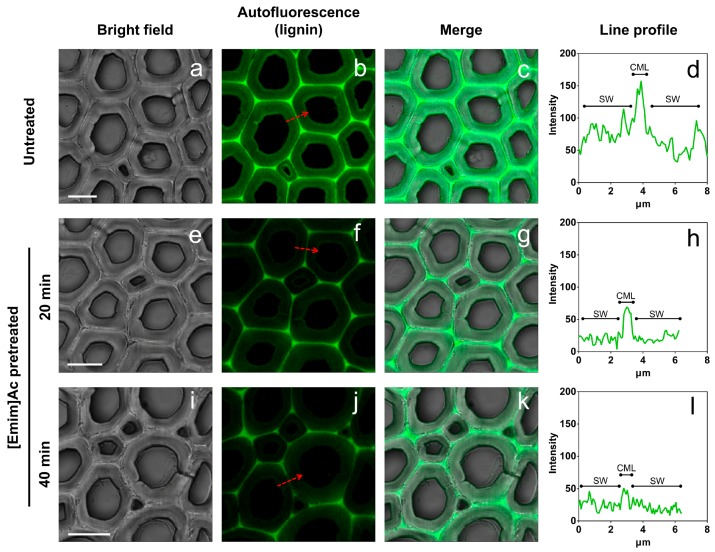
CLSM images of raw and [Emim]Ac pretreated poplar wood sections at 90 °C. (**a**–**d**) Untreated poplar; (**e**–**h**) IL pretreated poplar for 20 min; (**i**–**l**) IL pretreated poplar for 40 min. Arrows in (**b**,**f**,**j**) indicate the regions of line analysis. Scalebar = 10 μm.

To illustrate the morphological changes of poplar samples after [Emim]Ac pretreatment, field emission scanning electron microscopy (FE-SEM) and bright field microscopy imaging were performed on poplar flour and cross sections. The obtained FE-SEM and bright field microscopy images are shown in Figure 4 and Figure 5, respectively.

FE-SEM imaging technology revealed significant gross morphological changes in the IL pretreated poplar samples. Compared with the densified smooth surface of raw wood particles (Figure 4a,d), the pretreated samples showed a rougher more porous surface (Figure 4b,c,e,f, as shown by arrows), possibly caused by reversible swelling of the cellulose and the removal of lignin. These morphological changes increased the cellulose accessibility of the poplar samples, which was attributed to the improvement of enzymatic hydrolysis. The FE-SEM and bright field microscopy images of poplar cross sections before and after IL pretreatment illustrate the micromorphological changes at the subcellular level. The morphology of different cell wall lamellas can be observed. As can be seen from Figure 4g and Figure 5a, the cell walls of raw poplar samples were intact and flat; the compound middle lamella (CML) and the secondary wall (SW) kept close enough together. After pretreatment, the detachment between lamellas was observed; gaps between the CML and SW appeared (arrows in Figure 4h,i). This structural disruption of cell walls can increase the accessible surface area of cellulose, thus enhancing the sequential enzymatic hydrolysis. Lucas et al. [30] studied the reversible swelling of poplar cell walls during [Emim]Ac pretreatment at room temperature. In the FE-SEM images, the swelling of cell walls was not obvious (or observed), which may be attributed to the reshrinking of cell walls during drying. The increase of accessible surface area or porosity of biomass after IL pretreatment can enhance enzymatic hydrolysis; additionally, this may improve the sequential pretreatment efficacy in the combined pretreatment methods, such as two-step pretreatment with IL and alkali [31].

### 2.5. Topochemical Changes

Chemical composition distribution in wood cell walls is another significant factor affecting the cellulose accessibility. CLSM and CRM technologies were often used to visualize the components’ distribution in cell walls before and after IL pretreatment [14,21]. In this study, we performed CLSM and CRM to reveal the topochemical changes (lignin and carbohydrates distribution) in different lamellas of cell walls during [Emim]Ac pretreatment.

As can be seen from Figure 5, CLSM images were obtained to investigate the lignin distribution in poplar cross sections. Lignin is a complex heteropolymer with strong auto-fluorescence in the visible as well as far-IR regions [32]. The relative amount of lignin was illustrated by the fluorescence intensity in images. Prior to IL pretreatment, the spatial mapping of lignin distribution in sectioned poplar samples was accomplished using intrinsic lignin auto-fluorescence (Figure 5b). The CML regions have stronger fluorescence intensity, indicating this lamella had a higher degree of lignification. The fluorescence intensity of SW regions with higher concentrations of cellulose was lower. After IL pretreatment, the decline of fluorescence intensity in the whole images was observed (Figure 5f,j). This indicates the partial removal of lignin during pretreatment, which is coincident with chemical composition analysis. Moreover, the extent of the intensity decrease in the SW regions was different from that in the CML regions. In order to accurately quantify the intensity of fluorescence and further analyze the intensity changes after IL pretreatment, we performed line analysis. The fluorescence intensity on the line through the CML, as shown arrows in Figure 5b,f,j, was presented in Figure 5d,h,l, respectively. As we can see from the line profile, the center of CML region had a peak value of fluorescence before and after pretreatment. As expected, the image intensity decreased after [Emim]Ac treatment, due to the decline of lignin concentration. The intensity decrease from the raw sample to the IL pretreated sample for 20 min was significant; however the intensity changed slightly for IL pretreated samples from 20 min to 40 min. As a result, the cellulose conversion of poplar samples pretreated for 20 and 40 min was similar (69.02% and 73.64%, respectively; shown in Figure 1) and was dramatically higher than that of raw samples.

CRM is another technology used to determine the topochemical changes of plant cell walls during pretreatment [33]. In this study, we obtained Raman spectra and performed chemical imaging to investigate the effect of IL pretreatment on component distribution and concentration of plant cell walls over a large area.

**Figure 6:**
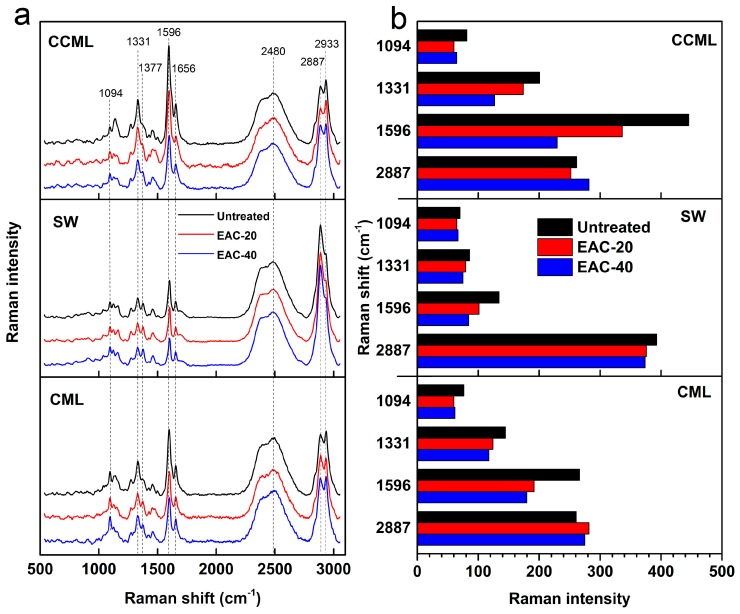
(**a**) Average Raman spectra for the CCML, SW, and CML regions of raw and [Emim]Ac pretreated poplar wood sections at 90 °C; (**b**) Variation of Raman intensity at four bands corresponding to lignin and carbohydrate concentration in the CCML, SW, and CML.

Figure 6a shows the average Raman spectra from the cell corner middle lamella (CCML), CML, and SW of poplar cell walls untreated and pretreated with [Emim]Ac at 90 °C for 20 and 40 min. The spectral range from 550 to 3150 cm^−1^, which includes bands from the wood components such as cellulose, hemicelluloses, and lignin, is of interest. There is little difference between the Raman spectral features of cellulose and hemicelluloses; the hemicelluloses bands are broader and can reside beneath the cellulose bands as a result of their low amount and amorphous character [34]. Therefore, the bands from cellulose and hemicelluloses cannot be distinguished and we refer to them as carbohydrates in this study. The bands at 1096, 1377, and 2887 cm^−1^ are assigned to C–O–C stretch (asymmetric), HCC, HCO, HOC bend, and C–H, C–H_2_ stretch, respectively. These mainly originate from carbohydrates. The bands at 1331, 1596, 1656, and 2933 cm^−1^, mainly from lignin, are assigned to aliphatic O–H bend, aromatic ring stretch (symmetric), C=C stretch of coniferyl alcohol, and C–H stretch in O–CH_3_, respectively [35,36]. As shown by the black line in Figure 6a, the prominent Raman peaks with high intensity for untreated poplar cell walls were observed. For the CCML region, the Raman intensity of lignin bands was higher than the CML and SW regions, which demonstrated the highest concentration of lignin in the CCML region. The SW region, however, contained more carbohydrates than other regions. After pretreatment with [Emim]Ac at 90 °C, we observed band intensity changes at all Raman bands for the whole cell wall area; however, the degree of changes in intensity of these bands differed across the three cell wall regions. To further quantify the discrepancy of intensity changes, we illustrated the Raman intensity of specific bands (typical characteristic lignin and carbohydrate bands) by bar diagram (Figure 6b). As can be seen from the lignin bands (1331 and 1596 cm^−1^), the slope of intensity decrease for the CCML region is steeper than that for the CML and SW regions during IL pretreatment. This indicated that the lignin concentration decreased dramatically for the CCML region as a result of [Emim]Ac pretreatment. However, the carbohydrate concentration changed slightly for all the cell wall regions.

**Figure 7:**
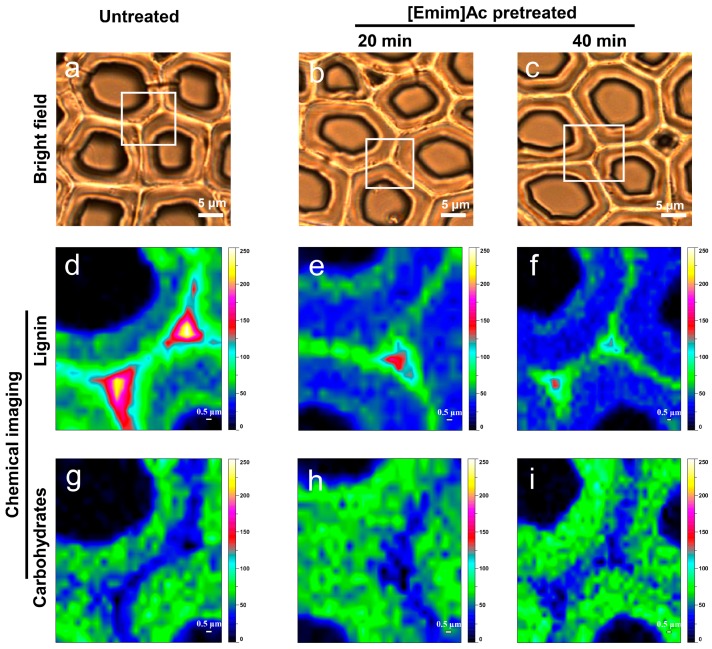
Raman mapping of poplar wood cell walls before and after [Emim]Ac pretreatment at 90 °C. Rectangles in bright field images (**a**–**c**) indicate the selected areas of mapping; (**d**–**f**) Lignin maps for untreated and IL pretreated poplar; (**g**–**i**) Carbohydrates maps for untreated and IL pretreated poplar.

To obtain more information about chemical variation in the cell walls over a large area during IL pretreatment and to enhance visualization of composition (i.e., lignin and carbohydrates) distribution, we used the Raman mapping technique. Chemical images of poplar cell walls obtained by CRM are presented in Figure 7. The cross sections untreated and pretreated with [Emim]Ac for 20 and 40 min were investigated. Rectangles in bright field images (Figure 7a–c) represent the selected areas of mapping. Raman images of lignin distribution (Figure 7d–f) were generated using a combined band region of 1570–1630 cm^−1^ and the sloping baseline method. As we can see, the image intensity decreased significantly for IL pretreated samples, which indicates the removal of lignin. Consistent with the results of average Raman spectra, although more lignin was removed from the CCML region, the decline of lignin concentration in the SW region was also remarkable. The band region of 2867–2907 cm^−1^ was selected to investigate the carbohydrate distribution, combing with the sloping baseline method. As shown in Figure 7g–i, the image intensity changed slightly during pretreatment, demonstrating the relative stability of carbohydrate concentration, which is consistent with the results of average Raman spectra and the above chemical composition analysis.

The delignification of poplar wood greatly increased the yield of enzymatic hydrolysis, because lignin reduces enzyme effectiveness by limiting the cellulose accessibility as well as by binding cellulase unproductively [37]. However, the relative contribution of these two roles of lignin in this study is not yet fully understood.

## 3. Materials and Methods

### 3.1. Materials

The dry poplar stems about 6 years of age used in this work were collected from the botanical garden of Beijing Forestry University, China. The raw material was knife-milled, and the wood flour (60–80 mesh) was extracted with toluene–ethanol (2:1, *v*/*v*) in a Soxhlet apparatus for 6 h. Then, the extractive-free sample was dried for 16 h in an oven at 60 °C for further use. For microscopic measurements, 10 μm-thick cross sections were cut from the secondary xylem of the dry poplar stems by a sliding microtome. The IL 1-ethyl-3-methylimidazolium acetate ([Emim]Ac) was purchased from the Lanzhou Institute of Chemical Physics, Lanzhou, China. The commercial cellulase used in this study was purchased from Novozyme, Bagsvaerd, Denmark, and the filter paper activity of the cellulase was 145 FPU/g.

### 3.2. IL Pretreatment

Poplar wood flour was prepared in [Emim]Ac at a concentration of 50 g/kg. After incubation at 90 °C under magnetic stirring for 20 and 40 min, the wood flour suspension was diluted with deionized water (at 10-fold higher mass than the IL) under stirring. Then, the suspended wood flour was recovered by filtration and washed with deionized water and dried in an oven at 80 °C for 2 h. The mass of recovered wood flour was then determined. Poplar wood sections were placed in 5 mL vials together with 1 mL of [Emim]Ac and were heated in an oven at 90 °C. After incubation for 20 and 40 min, the wood sections were rinsed with deionized water for 10–15 times to replace the residual IL from the samples.

### 3.3. Chemical Component Analysis

The chemical compositions of raw and pretreated poplar samples were determined according to the analytical procedures established by the National Renewable Energy Laboratory (NREL) [25]. The resulting solution was analyzed by a high-performance anion exchange chromatography (HPAEC) system (Dionex ICS 3000, Sunnyvale, CA, USA) with a pulsed amperometric detector, AS50 autosampler, the CarbopacTM PA-20 column (4 mm × 250 mm, Dionex, Sunnyvale, CA, USA), and the guard PA-20 column (3 mm × 30 mm, Dionex, Sunnyvale, CA, USA). All assays were performed in triplicate.

### 3.4. Enzymatic Hydrolysis

Enzymatic hydrolysis of the raw and pretreated poplar samples was carried out in a 25 mL digestion solution containing 0.05 M sodium acetate buffer (pH 4.8), using a 50 mL Erlenmeyer flask at a biomass loading of 2% (*w*/*v*). The cellulase was employed at the activity of 20 FPU/g substrate for all samples. The reaction mixture was digested in an air-shaking incubator at 48 °C and 150 rpm for 72 h. The IL pretreated poplar samples were rinsed with deionized water 10–15 times before enzymatic hydrolysis. To monitor hydrolysis kinetics, 100 µL of hydrolyzed slurry was taken periodically (0, 3, 6, 12, 24, 48, and 72 h) and analyzed by HPAEC. Results are presented as the percentage of the corresponding theoretical glucose yield of each sample. All assays were performed in triplicate.

### 3.5. Cellulase Adsorption Isotherm

To measure the cellulase adsorption isotherm, a range of cellulase concentrations (0.01–1.0 mg/mL) were incubated with the untreated and IL pretreated substrates, with a final dry solid content of 2% (*w*/*v*) in 50 mM acetate buffer (pH 4.8) at 4 °C for 90 min to reach equilibrium. After incubation, the supernatants were collected and centrifuged at 5000× *g* for 10 min. The free protein content of the supernatants was measured by Bradford protein assay (Tiangen, Beijing, China). The amount of cellulase bound to the substrate was indirectly calculated by subtracting the measured free protein from the total protein added. The experimental data were fit to the following Langmuir adsorption isotherm using software package OriginPro 8.0 (OriginLab, Northampton, MA, USA):

(1) $$E_{a}=\frac{E_{\max}K_{d}E_{f}}{1+K_{d}E_{f}}$$

where *E_a_* is the concentration of bound cellulase (mg/g substrate), *E*_max_ is the maximum content of absorbed cellulase (mg/g substrate), *E_f_* is the free protein concentration in the supernatant (mg/mL), and *K_d_* is the binding constant (mL/mg cellulase). Additionally, the distribution coefficient *(K_r_*) was calculated as follows:

(2) $$K_{r}=E_{max}\times K_{d}$$

### 3.6. Determination of Biomass Crystallinity

Crystallinity of raw and pretreated poplar samples was analyzed by powder X-ray diffraction in a D8 advance instrument (Bruker AXS, Karlsruhe, Germany), employing Ni-filtered Cu Kα radiation (wavelength = 0.154 nm) at 40 kV and 30 mA. Scans were obtained from 5° to 40° 2θ (Bragg angle) at 0.03° per second of scanning rate and at room temperature. Sample crystallinity, as expressed by CrI was measured from the XRD data and calculated with the following equation [38]:

(3) $$\mathrm{CrI}=\frac{I_{002}-I_{am}}{I_{002}}\times 100$$

where *I*_002_ is the scattered intensity for the crystalline portion of biomass at about 2θ = 22.5° and *I*_am_ is the intensity for the amorphous portion at about 2θ = 16.6°.

### 3.7. Field Emission Scanning Electron Microscopy

The surface morphological features of raw and pretreated poplar samples were studied by an FE-SEM apparatus (Hitachi S-4300, Tokyo, Japan) at an accelerating voltage of 5 to 10 kV. The wood flour particles and wood sections before and after IL pretreatment were prepared by vacuum drying. Subsequently, they were mounted on carbon tape-adhered aluminum stubs and sputter coated with 10–12 nm gold particles by using a vacuum sputter prior to acquiring images.

### 3.8. Confocal Laser Scanning Microscopy

The surface of raw and pretreated wood sections was observed by a confocal laser scanning microscope (Leica SP8, Wetzlar, Germany). An oil immersion objective lens (63 × NA = 1.40 in conjunction with the 10 × eye lens was used for observation, and a 488-nm argon laser was used for excitation [39]. Emission spectra were acquired at 500–570 nm for collection of the auto-fluorescence of lignin in wood samples. Image acquisition parameters were optimized initially and then kept constant for all samples. Digital analysis of CLSM images was conducted using the LAS AF Lite offline software (Leica, Wetzlar, Germany).

### 3.9. Confocal Raman Microscopy

The raw and pretreated wood sections were placed on a glass slide with a drop of D_2_O and then covered with a glass coverslip of 0.17 mm thickness for Raman detection. A LabRam Xplora exquisite full-automatic confocal Raman microscope (Horiba Jobin Yvon, Longjumeau, France) equipped with an MPlan 100× oil immersion microscope objective (Olympus, NA = 1.40) was utilized in this study. A linear polarized laser (diode-pumped green laser, λ = 532 nm), focused with a diffraction-limited spot size (0.61 λ/NA), was used to conduct measurements. The laser power on the sample was approximately 8 mW. The Raman light was detected by an air-cooled, front-illuminated, spectroscopic charge-coupled device (CCD) behind a grating (1200 grooves·mm^−1^) spectrometer. For mapping, an integration time of 2 s was chosen and every pixel corresponds to one scan with a spectrum acquired every 0.5 µm by averaging 2 s cycles. Labspec 5 software (Horiba Jobin Yvon, Longjumeau, France) was used for spectra analysis and image processing.

## 4. Conclusions

In summary, we have illustrated the cell wall deconstruction and lignin dissolution in poplar wood during [Emim]Ac pretreatment with short duration and low temperature, using chemical composition analysis, XRD, cellulase adsorption isotherm, and multiple microscopic techniques (FE-SEM, CLSM, and CRM). In these mild conditions, although decrystallization or crystallinity transformations were not observed, the enzymatic hydrolysis for the regenerated substrates was greatly improved compared to untreated samples, due to the increased cellulose accessibility and the decreased lignin-binding cellulase as a result of lignin removal. Additionally, the increase of porosity and chemical component dissolution may improve the effectiveness of second-step pretreatment in the integrated methods. The mild ILs pretreatment investigated in this study can not only enhance enzymatic hydrolysis with less energy input, but also provide a potential approach as the first step in improving the sequential pretreatment effectiveness.

## Figures and Tables

**Table 1:** Chemical composition of the poplar wood untreated and pretreated with [Emim]Ac.

Pretreatment ^a^		CrI (%) ^b^	Composition of the Residue (%) ^c^							
Time (min)	Residue Recovery (%)		Rha	Ara	Gal	Glu	Xyl	Man	AIL	ASL
Untreated	100	39.9	0.41	0.31	0.93	45.08	14.74	3.40	20.58	1.95
20	87.27	39.7	0.46	0.41	0.86	48.84	15.12	2.95	18.39	1.37
40	84.96	38.6	0.31	0.38	0.76	50.91	13.48	2.65	17.37	1.24

^a^ Two hundred and fifty milligrams of wood flour was incubated in 5 g [Emim]Ac under N_2_ with magnetic stirring at 90 °C for 20 and 40 min; ^b^ CrI represents the “crystallinity index”; ^c^ Determined by the NREL protocol [25]. Results are expressed as a percentage of the residues. All the measurements were obtained in triplicate, and the mean value has been indicated. Rha = Rhamnose, Ara = Arabian, Gal = Galactan, Glu = Glucan, Xyl = Xylan, Man = Mannan, AIL = Acid insoluble lignin, ASL = Acid soluble lignin.

**Table 2:** Langmuir isotherm parameters for cellulase binding to poplar wood untreated and pretreated with [Emim]Ac at 90 °C. EAC-20: [Emim]Ac pretreated for 20 min. EAC-40: [Emim]Ac pretreated for 40 min.

Substrate	Langmuir Isotherm Parameters		K_r_ (L/g)	R^2^
	E_max_ (mg/g)	K_d_ (mL/mg)		
Untreated	62.83	6.46	0.406	0.995
EAC-20	71.15	24.84	1.767	0.993
EAC-40	76.15	32.14	2.447	0.987
